# Injectable self-healing ceria-based nanocomposite hydrogel with ROS-scavenging activity for skin wound repair

**DOI:** 10.1093/rb/rbab074

**Published:** 2021-12-24

**Authors:** Xueyun Gong, Meng Luo, Min Wang, Wen Niu, Yidan Wang, Bo Lei

**Affiliations:** 1 School of Medicine, Henan Polytechnic University, Jiaozuo 454000, China; 2 Frontier Institute of Science and Technology, Xi’an Jiaotong University, Xi’an 710054, China

**Keywords:** bioactive materials, multifunctional scaffolds, rare earth dressing, wound healing

## Abstract

Excessive reactive oxygen species (ROS) in the injured skin may impede the wound repair and skin regeneration. Herein, we develop an injectable self-healing ceria-based nanocomposite hydrogel with ROS-scavenging activity to accelerate wound healing. The nanocomposite hydrogels were successfully prepared by coating cerium oxide nanorods with polyethylenimine and crosslinked with benzaldehyde-terminated F127 (F127-CHO) through the dynamic Schiff-base reaction (FVEC hydrogel). The results showed that the FVEC hydrogel possessed the good thermosensitivity, injectability, self-healing ability and ROS scavenging activity. The subcutaneous implantation experiments in mice confirmed that FVEC hydrogels are biocompatible and biodegradable *in vivo*. The full-thickness skin wound studies showed that FVEC hydrogel could significantly enhance the wound healing and epithelium regeneration with the formation of hair follicle and adipocyte tissue. This work provides a new strategy for the development of multifunctional Ce-based nanocomposite hydrogel for full-thickness skin wound healing and regeneration.

## Introduction

The rapid and efficient skin wound healing caused by the serious injury and full-thickness cutaneous defect are still the challenge in the field of regenerative medicine. In order to speed up the wound healing, appropriate wound dressings are usually used. The ideal wound dressing should have the following characteristics: (i) it has certain absorptive capacity for wound exudates; (ii) it has certain water-retention ability to provide a humid environment, which is helpful to maintain the activity of cells and enzymes [1]; and (iii) it can be closely fitted to the wound, convenient to use and easy to remove, so as to avoid secondary injury during replacement. Biomedical hydrogels have exactly these characteristics, so they have gained wide attention as the artificial dressing for wound healing. Biomedical hydrogel possesses a three-dimensional network containing water which are cross-linked by physical action or covalent bond by natural or synthetic polymers, and the structure was similar to the extracellular matrix of human body. Nanocomposite hydrogels with superior performance and customized function have been attracted the much attention in wound healing, which can be obtained by introducing nanomaterials into the three-dimensional network structure of hydrogels by physical embedding or chemical crosslinking. The structure and functions of hydrogels could be facilely controlled by the nanomaterials and polymer matrix. Due to the superior physical, chemical, electrical and biological properties, nanocomposite hydrogels have become one of the hot spots in biomedical field and have a broad application prospect in wound healing and tissue regeneration [2–9].

Skin damage causes inflammation, which leads to the accumulation of immune cells at the wound site and the release of reactive oxygen species (ROS) [10]. Low level of ROS is an essential substance for a variety of signal transduction pathways in organisms. It can also resist the invasion of bacteria and other pathogens and play an active regulatory role in wound repair [11]. Studies have shown that low levels of ROS can stimulate cell migration and promote angiogenesis [12, 13]. However, excessive production of ROS will lead to oxidative stress, cause cell damage, delay the transition of wound site from the inflammatory stage to the proliferative stage and lead to slow or even difficult wound healing [14]. Therefore, regulating the redox balance of the microenvironment at the wound site to avoid oxidative stress and ensuring the normal growth of cells is crucial to wound healing. The appropriate use of antioxidants to regulate the redox microenvironment at the wound site has been shown to be an effective way to improve the wound repair process [15]. Some antioxidants such as puerarin, conductive polymer, gallic acid and curcumin have been introduced into hydrogels to prepare antioxidant hydrogels [16–19]. Cerium oxide has a good scavenging capacity of ROS, which can scavenging hydroxyl radical [20], superoxide anion and so on [21] and protect cells from oxidative stress. As an antioxidant, cerium oxide can exert an anti-inflammatory effect by reducing the production of NO [22]. It can also reduce apoptosis by inhibiting ROS concentration to prevent retinal degeneration [23]. Therefore, it is very interesting to design Ce-based nanocomposites hydrogel dressing for wound healing and skin regeneration.

**Figure 1.**
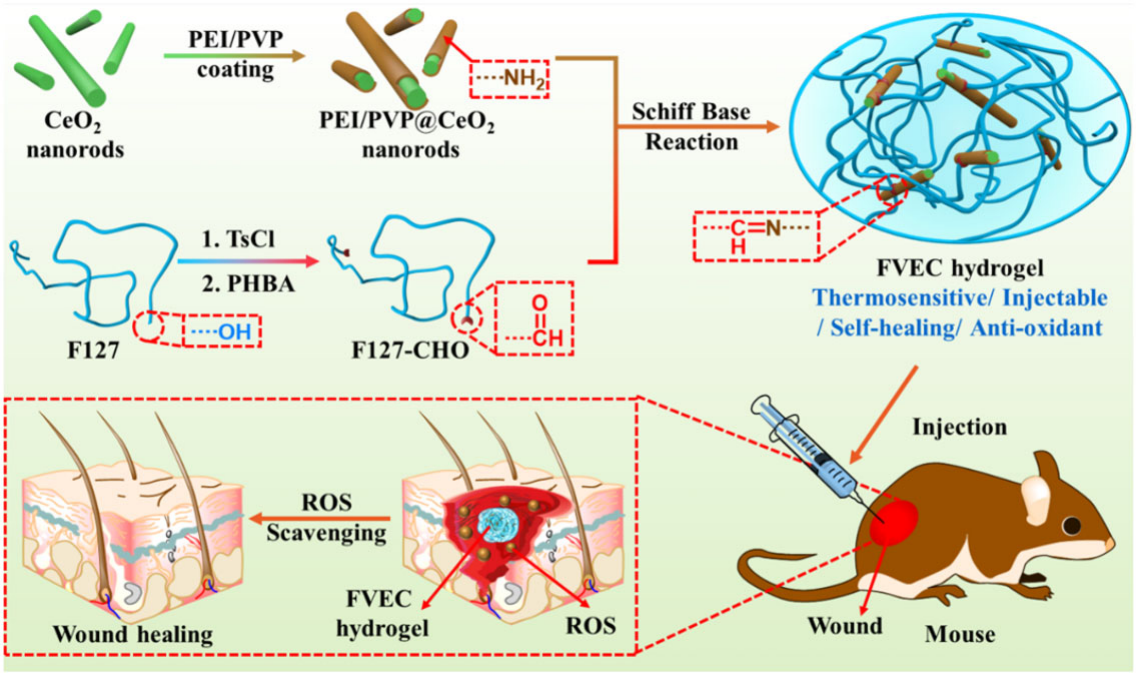
Schematic illustration of design and wound healing application of multifunctional bioactive Ce-based FVEC hydrogel.

In this article, we combine the unique ROS-scavenging ability of cerium oxide nanorods with the advantages of hydrogel and prepare a kind of antioxidant nanocomposite hydrogel with temperature sensitivity, injectable and self-healing properties. Pluronic F127 (F127) is a Food and Drug Administration-approved polymer that can be used *in vivo* in the biomedical field [24]. It is widely used as a drug carrier because of its good biocompatibility and temperature-responsive gelation behavior [25, 26]. Here, the nanocomposites hydrogel (FVEC) was fabricated by the double networks composed of F127-CHO and PEI/PVP@CeO_2_. As shown in Fig. 1, the F127-CHO formed the first thermosensitive network and the dynamic crosslinking between F127-CHO and PEI/PVP@CeO_2_ was the second network. The cerium oxide nanorods modified with amino groups are doped in the hydrogel and form dynamic Schiff-base bonds with the aldehyde groups on F127-CHO. The FVEC nanocomposite hydrogel has the multifunctional properties of thermosensitivity, injectability, self-healing and excellent scavenging ability of ROS. The effect of FVEC hydrogel on the wound healing was further investigated using a full-thickness skin defect in the mice.

## Materials and methods

### Fabrication and characterization of FVEC hydrogel

The CeO_2_ nanorods were prepared in a similar way to literature method [27]. The morphology and structure were characterized and confirmed by transmission electron microscopy (TEM) (H-7700, Hitachi) and X-ray diffraction spectroscopy (XRD) (Rigaku D/MAX-RB). The details of synthesis process are shown in supporting information (SI). The PEI/PVP@ CeO_2_ was prepared as follows. Briefly, 0.1345 g of CeO_2_ nanorods were ultrasonically dispersed in deionized water, and then an aqueous solution containing 0.6 g of polyvinyl pyrrolidone (PVP) and 0.3 g of polyethyleneimine (PEI) were added dropwise to the aqueous solution of CeO_2_, stirred at room temperature for 2 h, centrifuged and freeze-dried to obtain CeO_2_ nanorods coated with PEI and PVP, denoted as PEI/PVP@ CeO_2_. The morphology and structure were characterized by TEM and Fourier transform infrared (FT-IR) spectrometer (NICOLET 6700, Thermo). F127-CHO was synthesized with reference to the literature, and its structure was determined by ^1^H nuclear magnetic resonance spectroscopy (^1^H NMR) (AvanceTM 400, Bruker) (details in SI). At 0°C, a certain amount of PEI/PVP@CeO_2_ nanorods were added to the mixed aqueous solution of F127 and F127-CHO with a mass ratio of 7–3, so that the final mass ratio of PEI/PVP@CeO_2_ was 0 wt%, 0.5 wt%, 1.0 wt% and 2.0 wt%. The mixture was stirred evenly and placed in an oven at 37°C to obtain hydrogels, which were named FVEC-0, FVEC-1, FVEC-2 and FVEC-3, respectively. The morphology and structure of the hydrogels were characterized by scanning electron microscopy (SEM) (GeminiSEM 500, Zeiss), XRD and FT-IR.

### Rheological and multifunctional properties evaluations

The thermal sensitivity of the nanocomposite hydrogel was investigated by observing its macroscopic state at three representative temperature of 4°C, 25°C and 37°C. The self-healing ability of the nanocomposite hydrogel was evaluated by observing the fusion state of two separated hydrogels of different colors after being put together. Its injectability was evaluated by placing the hydrogel in a medical syringe and then injecting it through the needle. The TA rheometer (DHR-2) was used to measure the modulus of the nanocomposite hydrogel at different temperatures and oscillating strains and the viscosity change at different shear rates to investigate its rheological properties.

### Evaluation of antioxidant performance

The ability of hydrogel to scavenging hydroxyl radicals was investigated by UV-visible spectrophotometry. Methyl violet (MV), FeSO_4_, H_2_O_2_, Tris-HCl buffer and hydrogel solutions with different contents were prepared. The absorption spectra and peak absorption were determined by a UV-visible spectrophotometer (Lambda 950, PerkinElmer) after incubation for 10 min at room temperature. The method of solution configuration and determination and the results are shown in SI. Superoxide dismutase (SOD) analysis kit method (WST-1) was used to determine the ability of hydrogel to remove superoxide anions. The hydrogel containing 0, 0.01, 0.03, 0.06, 0.09, 0.12 mM PEI/PVP@CeO_2_ and analytical solutions containing xanthine, xanthine oxidase and WST-1 were incubated at 37°C for 20 min. Then the absorption value at the wavelength of 450 nm was measured by a microplate reader.

### Biodegradation evaluation *in vitro* and *in vivo*

*In vitro* degradation of nanocomposite hydrogels was carried out in phosphate buffer solutions at pH 5.5 and pH 7.4. Briefly, 200 µl of nanocomposite hydrogel was prepared in a small disc, which was placed in a 24-well plate with 1 ml of buffer solution and then placed in a 37°C oven. Every 24 h, the small disc was taken out, dried at 37°C and weighed, while the buffer was replaced by fresh buffer. Degradation of hydrogels was carried out at 37°C, and five samples were tested in parallel. The degradation of hydrogels was investigated by weightlessness. Five replicates of each sample were measured in parallel.

*In vivo* degradation was carried out by injecting hydrogels subcutaneously into the backs of mice. Briefly, the mice were anesthetized, then the skin on the back was gently pinched and 200 µl of hydrogel was injected subcutaneously with a medical syringe. After implantation of 0, 1, 2, 3, 5 and 7 days, the mice were sacrificed. The implanted hydrogel and surrounding tissues were cut off together with the skin to observe the status of the hydrogel. The sample tissues after 1, 3 and 7 days of implantation were sectioned for hematoxylin-eosin (H&E) analysis. The degradation of hydrogel *in vivo* was observed with an optical microscope (BX53, Olympus). All animal experiments were approved by the Animal Committee of Wenzhou Medical University.

### Cytotoxicity and hemocompatibility assay

The cytotoxicity of hydrogel was investigated by incubating the material with fibroblast L929. Live/dead kit and Alamar blue kit (Invitrogen) were used for evaluation. The blood compatibility of hydrogels was evaluated by hemolysis test. The experimental method and procedure are shown in SI.

### Cutaneous wound-healing examination

The skin wound-healing experiment was conducted on female mice weighing 30–35 g. All animal experiments were approved by the Animal Committee of Wenzhou Medical University. The mice were randomly divided into four groups. After anesthesia, round skin with a diameter of 7 mm was removed from the back of the mice. The wound of the first group was covered with 3M Tegaderm film. The second group of mice was untreated. The third group of mice was coated the wound with hydrogel FVEC-0 without PEI/PVP@CeO_2_, while the fourth group of mice was coated the wound with nanocomposite hydrogel FVEC-1. The wound healing was observed and recorded. After 0, 3, 8 and 14 days, the wound was photographed and the wound area was calculated by Image J. The mice were sacrificed 3, 8 and 14 days later, the wound sites and its surroundings were cut off, H&E staining was performed and the tissue sections were observed with an inverted microscope (IX53, Olympus).

### Statistical analysis

All experimental quantitative data were shown as means and standard deviation. The statistical assay was carried out using Student’s *t*-test, and the statistically significant difference was considered when *P <* *0.05 and *P <* *0.01.

## Results and discussion

### Fabrication and characterization of FVEC hydrogel

**Figure 2.**
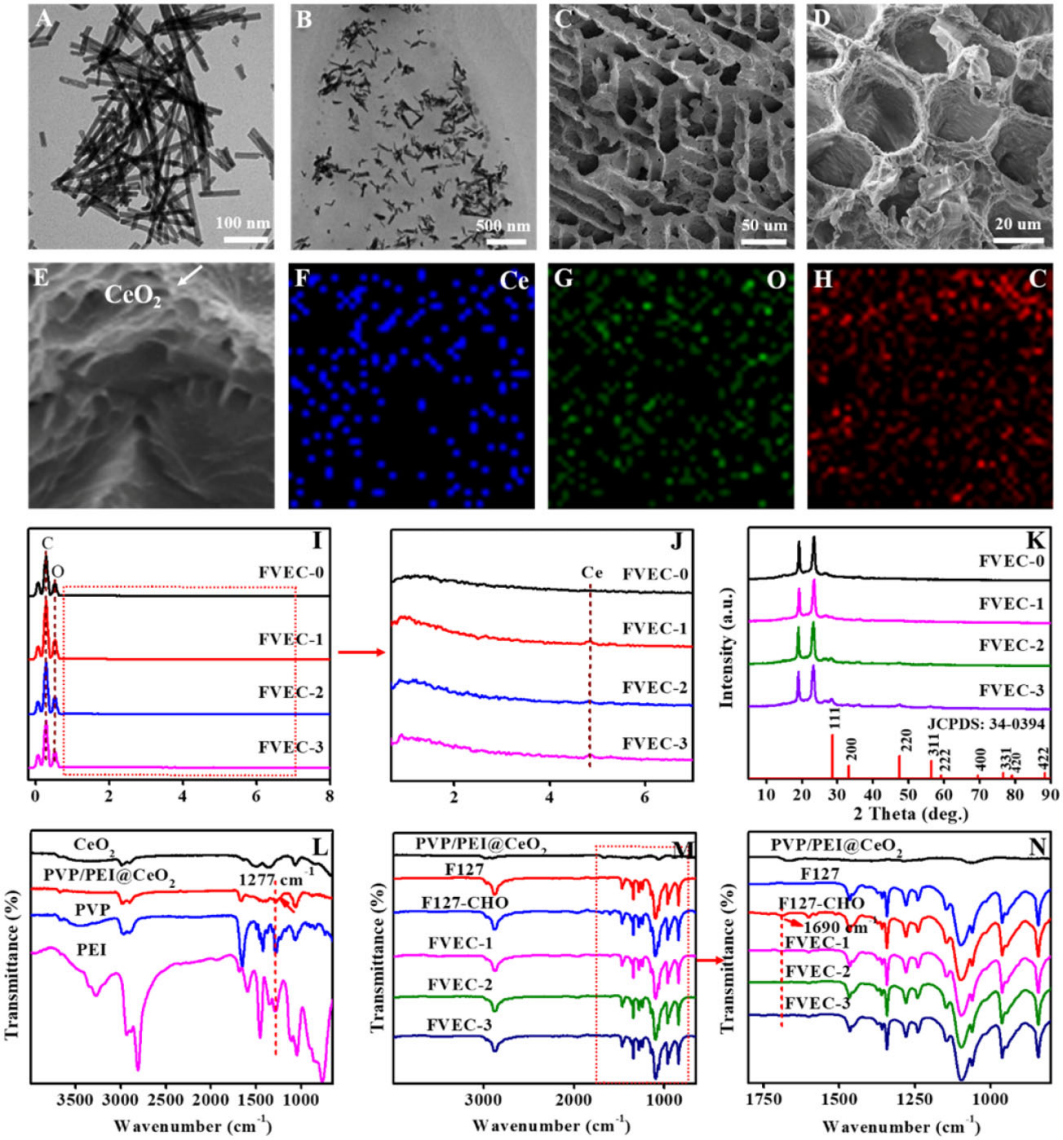
Physicochemical structure characterizations. (**A**) TEM images of PVP/PEI@CeO_2_; (**B**) TEM images of nanocomposite hydrogel; (**C**–**E**) SEM images of nanocomposite hydrogel; (**F**–**H**) element mapping of nanocomposite hydrogel; (**I**–**G**) EDS spectra; (**K**) XRD patterns; and (**L**–**M**) FT-IR spectra; (**N**) Enlarged view of the part in the red dashed box in M.

Figure 1 shows the fabrication process and potential application in wound healing of FVEC hydrogel. Figure 2 exhibits the structure characterizations of samples. After reaction with PVP and PEI, PEI/PVP @CeO_2_ nanorods (Fig. 2A) became shorter than pure CeO_2_ nanorods (Supplementary Fig. S1A). The morphology of the FVEC nanocomposite hydrogel is shown in Fig. 2B–D. From TEM images (Fig. 2B), it was seen that CeO_2_ nanorods are distributed in the hydrogel matrix. According to SEM images (Fig. 2C and D), FVEC hydrogel has a typical three-dimensional porous structure. The typical CeO_2_ nanoparticles could been in the hydrogel (Fig. 2E). Element mapping (Fig. 2F–H) also confirmed the presence of Ce in the composite hydrogel. EDS results (Fig. 2I and J) show that Ce element does not exist in the hydrogel FVEC-0 formed by pure F127 and F127-CHO, while the content of PEI/PVP@CeO_2_ nanorods increased from 0.5 wt% to 1.0 wt% and 2.0 wt%, the peak intensity of Ce element increased in FVEC-1, FVEC-2 and FVEC-3. XRD analysis results show that with the increase of PEI/PVP@CeO_2_ content, the characteristic diffraction peak of CeO_2_ (JCPDS: 34-0394) appeared in the nanocomposite hydrogel (Fig. 2K). The chemical structures of PVP, PEI, CeO_2_ and PVP/PEI@CeO_2_ were determined by FT-IR spectroscopy (Fig. 2L). It could be seen that compared with pure CeO_2_, a new absorption peak appeared at 1277 cm^−1^ in PVP/PEI@CeO_2_, which was the stretching vibration of C-N, indicating that PVP/PEI was coated on CeO_2_ nanorods. The structures of F127, F127-CHO, PVP/PEI@CeO_2_ and FVEC-1, FVEC-2 and FVEC-3 hydrogels were also determined by FT-IR spectroscopy (Fig. 2M and N). Compared with F127-CHO, the disappearance of the peak at 1690 cm^−1^ in the infrared spectra of FVEC-1, FVEC-2 and FVEC-3 indicated that the Schiff-base reaction between F127-CHO and PVP/PEI@CeO_2_ was occurred (Fig. 2N). There was no significant difference between FVEC-1, FVEC-2 and FVEC-3 hydrogel on the FTIR spectrum.

### Multifunctional properties evaluation of nanocomposite hydrogels

**Figure 3.**
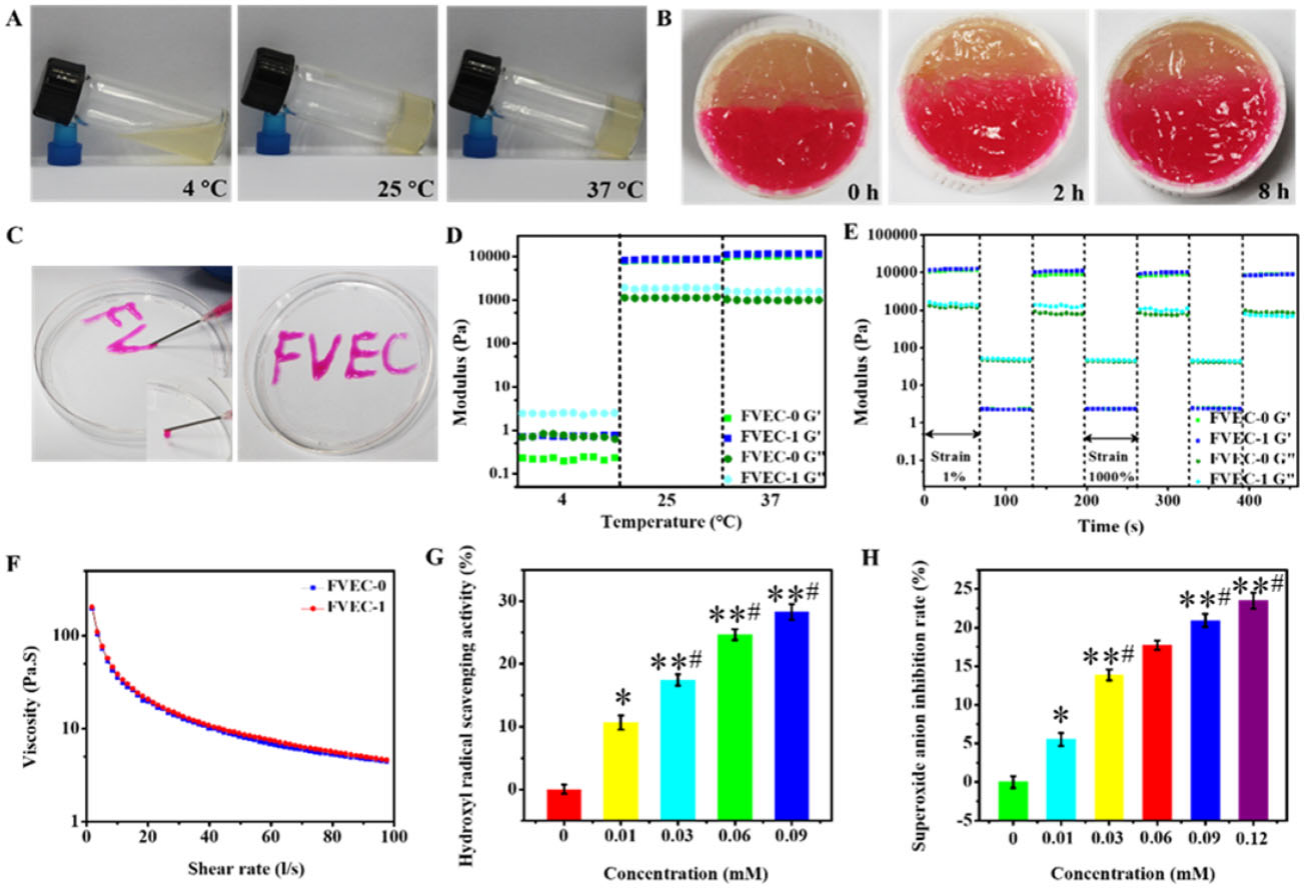
Multifunctional properties of FVEC nanocomposite hydrogel. (**A**) Optical pictures of nanocomposite hydrogel at 4°C, 25°C and 37°C. (**B**) Images of self-healing of nanocomposite hydrogel. (**C**) Images of injectability of nanocomposite hydrogel. (**D**) G′ and G″ of FVEC hydrogel at 4°C, 25°C and 37°C. (**E**) G′ and G″ of FVEC hydrogel when the step strain switched from 1% to 1000% at 37°C. (**F**) Viscosity changes of FVEC hydrogels at different shear rates. (**G**) Hydroxyl radical clearance of FVEC hydrogels at different CeO_2_ concentrations. (**H**) Superoxide anion clearance of FVEC hydrogels at different CeO_2_ concentrations. *P <* *0.05 relative to 0 mM, *^#^P *<* *0.05 relative to 0.01 mM, **P <* *0.01 relative to 0 mM.

Considering that CeO_2_ nanomaterials were difficult to be degradable *in vivo* and in wound, the FVEC-1 hydrogel with low content of CeO_2_ nanorods was chosen as the example to evaluate their properties and the role in wound healing. Figure 3 shows the results of thermal sensitivity, self-healing ability and injectability of FVEC-1 hydrogel. It could be seen from the state of the hydrogels in the three small glass bottles tilted at different temperatures in Fig. 3A that the hydrogels presented a solution state at low temperature (4°C), and a gel state at 25°C and 37°C. To test the self-healing ability of hydrogel, two semicircular nanocomposite hydrogels with different colors were placed together at 37°C and the self-healing situation was observed. As seen from the photos taken, the interface between the two gels gradually disappeared over time, and the two gels gradually fused together (Fig. 3B), indicating the good self-healing ability which may be due to the existence of dynamic Schiff-bonds in the nanocomposite hydrogel. Figure 3C shows the good injectability of the hydrogel. The hydrogel can be freely drawn from the needle of the medical syringe and the desired letter can be written. The thermosensitivity, self-healing and injectable behavior of hydrogel could be supported by the rheological analysis. It was seen from the modulus diagram at different temperatures that the loss modulus G″ of the nanocomposite hydrogel is greater than the energy storage modulus G′ at 4°C, while at 25°C and 37°C, the energy storage modulus G′ is greater than the loss modulus G″ (Fig. 3D). It could be seen from the modulus diagram of the nanocomposite hydrogel under different strains that under high strain conditions (1000%), the storage modulus G′ and the loss modulus G″ decrease sharply, but after three cycles of strain from 1% to 1000%, the storage modulus G′ and loss modulus G″ could still be restored to their original values (Fig. 3E), suggesting their good self-recovery behavior. The viscosity of the hydrogel decreased sharply as the shear rate increased from 0 l/s to 100 l/s, indicating that the hydrogel has typical shear thinning ability and injectability (Fig. 3F). The results showed that there was no significant difference in rheological properties between FVEC-0 and FVEC-1 hydrogel, which was probably that the doped content of PEI/PVP@CeO_2_ was very low in the hydrogel.

The antioxidant properties of nanocomposite hydrogels were evaluated by two methods. Firstly, the scavenging ability of strong oxidant hydroxyl radical (·OH) was tested by spectrophotometry. Methyl violet (MV) has a characteristic absorption peak at 592 nm, and hydroxyl radical can react with it to reduce the absorbance at the maximum absorption. The decrease of absorbance is related to the level of hydroxyl radical, in which the adsorption intensity at 592 nm would increase when the hydroxyl radicals were scavenged. The presence of CeO_2_ in the nanocomposite hydrogel could protect the MV by competing with the hydroxyl radicals and slowed down the decrease in absorbance. Through the change of absorbance, the scavenging ability of hydrogel to ·OH could be investigated. In this experiment, the ·OH is derived from the classical Fenton reaction that the reaction between bivalent iron ion and hydrogen peroxide. The results are shown in Fig. 3G, Supplementary Fig. S3A and B. The single aqueous solution of FeSO_4_, H_2_O_2_ and FVEC-1 has no absorption at 592 nm (Supplementary Fig. S3A). The mixed solution of FeSO_4_ and H_2_O_2_ also had no absorption at 592 nm. Only the aqueous solution of MV presents a characteristic absorption peak at 592 nm. As shown in Supplementary Fig. S3B, the absorbance of the mixture of MV, H_2_O_2_ and nanocomposite hydrogel FVEC-1 at 592 nm was the same as that of the pure MV aqueous solution. The addition of nanocomposite hydrogel FVEC-1 has no effect on the absorption of MV. The mixture of MV, FeSO_4_ and H_2_O_2_ had a lower absorbance at 592 nm, which was due to the further reaction of ·OH produced by Fenton reaction with MV. However, the peak intensity of the mixture of MV, FeSO_4_, H_2_O_2_ and nanocomposite hydrogel FVEC-1 was significantly higher than that without FVEC-1, which further indicated the good antioxidant activity of FVEC-1 hydrogel against ·OH. In addition, the clearance rate of hydroxyl radical was significantly improved as the increase of FEVC-1 content (Fig. 3G). To further analyze the antioxidant activity, the time-dependent effect of hydrogel on ·OH was studied. As shown in Supplementary Fig. S4, the scavenging ability of ·OH was increased first and decreased after 10 min reaction. The H_2_O_2_ scavenging ability analysis of hydrogel was also performed (Supplementary Fig. S5). The new results showed that the nanocomposites hydrogel could efficiently scavenge the hydroxyl radical at 10 min and the H_2_O_2_ scavenging ability was not good. It should be noted that the ROS clearance efficiency of pure CeO_2_ was a little high compared with CeO_2_-contained hydrogel (Supplementary Fig. S6). We also used an SOD assay kit (WST-1) to determine the superoxide anions scavenging activity of the FVEC-1 nanocomposite hydrogel. The relationship between the inhibition rate of superoxide anion and the concentration of CeO_2_ in the system is shown in Fig. 3H, which also suggested that the superoxide anions scavenging activity depends on the content of FVEC-1 hydrogel. These results fully demonstrated that the FVEC-1 nanocomposite hydrogel has good antioxidant capacity.

### Evaluation of cytotoxicity and blood compatibility

**Figure 4.**
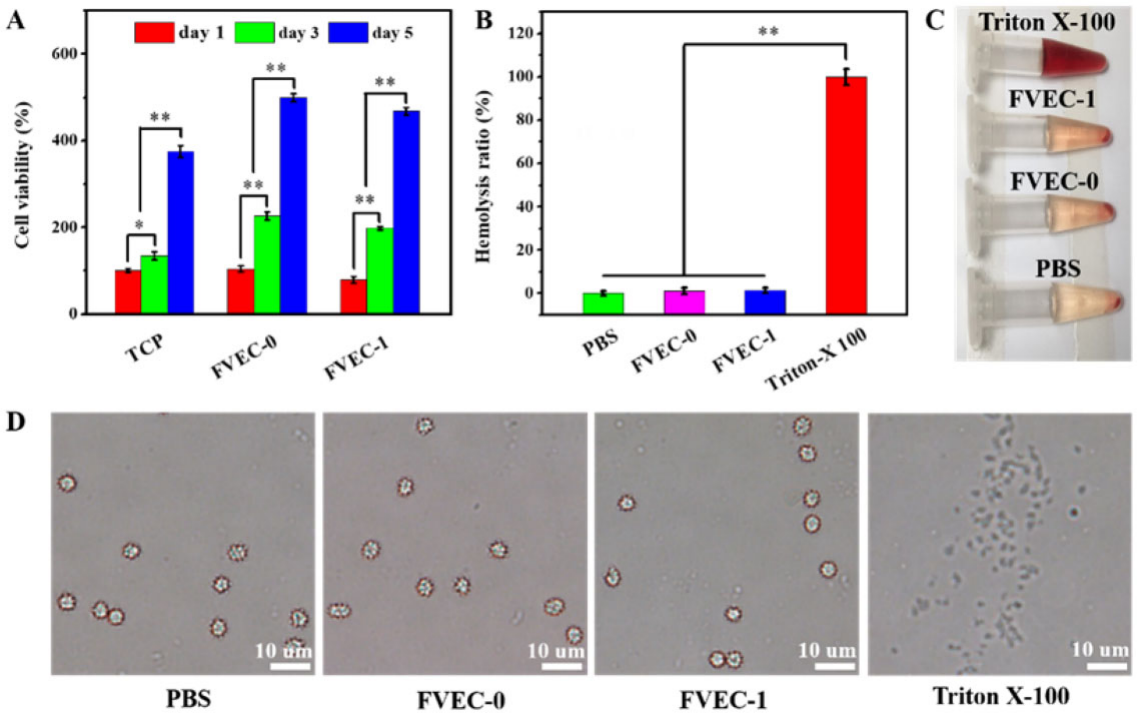
Cytotoxicity and blood compatibility analysis. (**A**) L929 cells viability after being cultured with FVEC-0 and FVEC-1 hydrogel for 1, 3 and 5 days (TCP as the control). (**B**) Hemolysis ratio analysis of RBCs after incubation with various samples. RBCs solution images (**C**) and microscope images (**D**) after being treated with PBS, FVEC-0, FVEC-1 and Triton-X100. *P <* *0.05 and **P <* *0.01.

The cytotoxicity and hemolytic test results of the nanocomposite hydrogel are shown in Fig. 4. After co-culturing hydrogel with fibroblast L929 for 1, 3 and 5 days, the cell viability was evaluated. As shown in Fig. 4A, the L929 cell viability was significantly increased as the increase of incubation time from 1 to 5 days in all groups. On day 1, day 3 or day 5, the cell viability in FVEC-0 and FVEC-1 groups was also significantly higher than the TCP group, suggesting that FVEC hydrogel had the good cell compatibility. The live/dead staining also indicated that most of cells in the FVEC-1 group were alive on day 5 with only a few red dead cells (Supplementary Fig. S7). The hemocompatibility analysis of FVEC hydrogel is shown in Fig. 4B and C. As shown in Fig. 4B, FVEC-0 and FVEC-1 hydrogel showed the very low hemolysis (below 5%), which was comparable to PBS and significantly low compared with Triton-X 100. Additionally, after incubation with FVEC-0 and FVEC-1 for 1 h, the morphology of the red blood cells did not change significantly and remained in a complete spherical shape, indicating that no obvious hemolysis occurred (Fig. 4C and D). The good cell compatibility and blood compatibility make the FVEC nanocomposite hydrogels suitable for *in vivo* wound-healing application.

### *In vitro* and *in vivo* degradation of nanocomposite hydrogel

**Figure 5.**
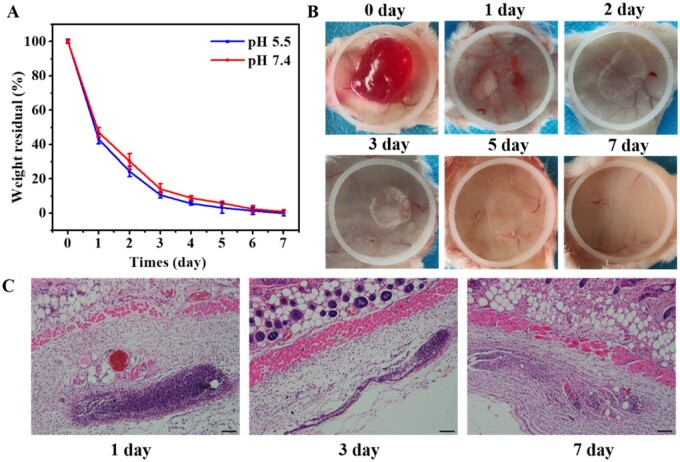
*In vitro* and *in vivo* degradation of FVEC nanocomposite hydrogel. (**A**) *In vitro* biodegradation in pH 5.5 and pH 7.4 buffer solutions. (**B**) Representative images of the nanocomposite hydrogel after implantation 0, 1, 2, 3, 5 and 7 days. (**C**) H&E staining of nanocomposite hydrogel with the surrounding tissue after 1, 3 and 7 days of implantation (scale bars: 50 µm).

The *in vitro* degradation of the FVEC nanocomposite hydrogel was performed in pH 5.5 and pH 7.4 buffer solutions, and the results are shown in Fig. 5A. After 7 days, the hydrogel was almost completely degraded at pH 7.4. In pH 5.5 medium, the degradation rate is slightly faster, which may be related to the fact that the Schiff-base bond is more likely to break under acidic conditions. The *in vivo* degradation of nanocomposite hydrogel was investigated by implanting FVEC-1 under the skin on the back of mice. At different time points (1, 2, 3, 5, 7 days) after implantation, it was removed to observe its macro morphology, and the results are shown in Fig. 5B and C. It could be seen that compared with the initial implanted hydrogel (0 day), the volume of the nanocomposite hydrogel was significantly reduced with the extension of the implantation time, and the existence of the hydrogel was hardly observed after 7 days (Fig. 5B). After 1, 3 and 7 days of implantation, the mice were sacrificed for histological examination. From the H&E staining results, it was found that after 1 day of implantation, most of the hydrogel was still in a dense state, with only a few defects (Fig. 5C). After 3 days of implantation, the hydrogel structure was relatively loose, and voids were formed in some areas, and the degradation was more obvious. On day 7 after implantation, the hydrogel was almost completely replaced by autologous fibrous tissue. Both the results *in vitro* and *in vivo* showed that the nanocomposite hydrogel was degradable, which can be explained by the structure of hydrogel. As a typical amphiphilic triblock copolymer, F127 could self-assemble into the micelles in water and this micelle would be disassembly *in vivo*. In addition, the amino group of PEI/PVP@CeO_2_ combined with the aldehyde group of F127-CHO to form Schiff-base bond, which could be broken *in vivo* environment. Both of these effects are dynamic, so the hydrogel can be degraded *in vitro* and *in vivo*.

### Cutaneous wound-healing evaluation *in vivo*

**Figure 6.**
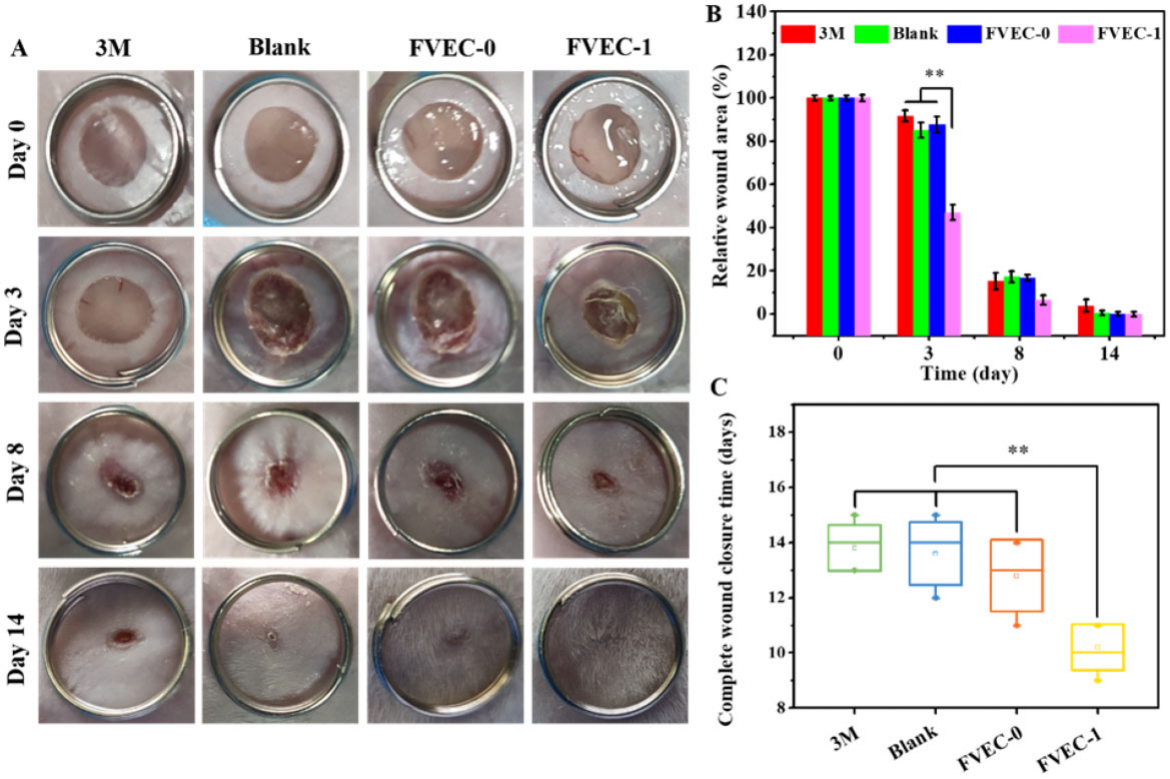
Macroscopic wound healing evaluation *in vivo*. (**A**) Representative skin wound photographs on days 0, 3, 8 and 14. (**B**) Relative wound area on days 0, 3, 8 and 14. (**C**) Wound closure time (*P <* *0.05, **P <* *0.01).

Based on the good thermosensitivity, injectability, self-healing, antioxidant activity and biodegradability, as well as the biocompatibility of FVEC hydrogel, we further evaluated their *in vivo* skin wound healing and tissue repair by a rat model. Although the antioxidant activity of hydrogel was increased as the improvement of CeO_2_ content, the high CeO_2_ content resulted in the non-biodegradation in the wound tissue, which was not benefitable to skin tissue regeneration. Therefore, in this study, the FVEC-1 with low CeO_2_ content was used to investigate the effect of wound healing *in vivo*, commercial 3M Tegaderm film, FVEC-0 group was served as a control. The representative time points selected were 3, 8 and 14 days, the macro treatment effect was observed and photographed, as shown in Fig. 6A. After 3 days, compared with the other three groups, the wound exudate in the FVEC-1 group was completely absorbed, and the wound area was significantly reduced (Fig. 6A and B). There was no significant difference in wound healing between 3M and Blank and FVEC-0 groups. After 8 days of treatment, the wound area of mice treated with FVEC-1 was significantly smaller. After 14 days, the wounds of mice treated with FVEC-1 were completely closed, the wound surface was covered with new skin, and the epidermal tissue was smooth, the other three groups still had scars, especially in the 3M group. The statistical results of wound area at different time points confirmed that the use of FVEC-1 hydrogel could reduce the wound area faster (Fig. 6B). In addition, the statistical results of the complete wound healing time showed that the FVEC-1 hydrogel group could significantly accelerate the wound healing speed and shorten the wound healing time, as compared to other control groups (Fig. 6C).

**Figure 7.**
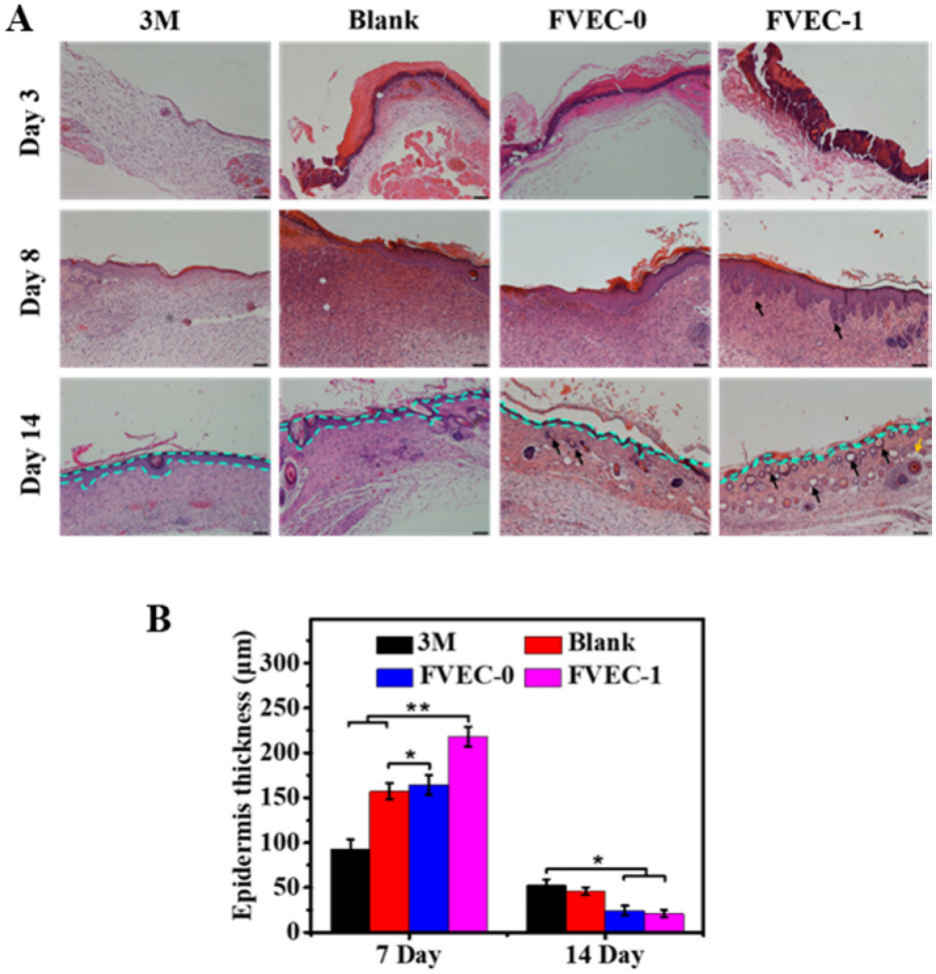
Histological evaluation of wound healing and skin regeneration after treated by various samples for 3, 8 and 14 days. (**A**) H&E staining tissue images (scale bar = 100 µm), green dotted lines indicate the epithelium border, black arrows present the hair follicles and yellow arrows show the adipose cells. (**B**) Statistical results of the epithelium thickness (*P <* *0.05, **P <* *0.01).

The healing effect of FVEC hydrogel on the skin wounds was further evaluated by the histological examination (H&E staining). As shown in Fig. 7, on day 3, compared with other groups, the FVEC-1 group showed the obvious blood crust which was helpful for further wound healing. On day 8, the newborn skin appendages such as hair follicles were observed in tissue sections in FVEC-1 group, while almost no hair follicles appeared in other groups. On day 14, a large number of hair follicles and some fat cells were observed in tissue sections in the FVEC-1 group, and some hair follicles also appeared in the FVEC-0 group (Fig. 7A). The statistical results on the epithelium thickness also showed that the FVEC-1 group could efficiently enhance the formation of epithelium tissue on day 7 and recover to the normal thickness like the native skin tissue, as compared to the control groups (Fig. 7B). The results of histological analysis further confirmed that FVEC-1 hydrogel could effectively enhance the wound healing and skin tissue regeneration.

## Conclusion

In summary, we developed a novel antioxidant cerium-based FVEC nanocomposite hydrogel with multifunctional properties for skin wound repair. The FVEC nanocomposite hydrogel has the characteristics of temperature sensitivity, injectability and self-healing. In addition, the nanocomposite hydrogel also has biodegradability and good biocompatibility. It can effectively remove ROS and can significantly accelerate the wound closure speed, promote the wound healing and tissue formation. This study provides a new strategy for designing anti-oxidation hydrogels with multifunctional properties for enhanced wound healing and skin regeneration.

## Supplementary data

Supplementary data are available at *REGBIO* online.

## Funding

This work was supported by the Scientific and Technological Project of Henan Province (182102310300) and Key Research Projects of Colleges and Universities in Henan Province (18A150028).

*Conflict of interest statement*. None declared. 

## Supplementary Material

rbab074_Supplementary_Data

## References

[1] Xiao Y , Reis LA, Feric N, Knee EJ, Gu J, Cao S, Laschinger C, Londono C, Antolovich J, McGuigan AP, Radisic M. Diabetic wound regeneration using peptide-modified hydrogels to target re-epithelialization. Proc Natl Acad Sci USA 2016;113:5792–801. 10.1073/pnas.1612277113 PMC5056062 27647919

[2] Wang X , Chang Ji, Wu C. Bioactive inorganic/organic nanocomposites for wound healing. Appl Mater Today 2018;11:308–19.

[3] Motealleh A , Kehr NS. Nanocomposite hydrogels and their applications in tissue engineering. Adv Healthcare Mater 2017;6:1600938. 10.1002/adhm.201600938 27900856

[4] Thoniyot P , Tan MJ, Karim AA, Young DJ, Loh XJ. Nanoparticle-hydrogel composites: concept, design, and applications of these promising, multi‐functional materials. Adv Sci (Weinh) 2015;2:1400010. 27980900 10.1002/advs.201400010 PMC5115280

[5] Zhou L , Xi Y, Xue Y, Wang M, Liu Y, Guo Y, Lei B. Injectable self‐healing antibacterial bioactive polypeptide‐based hybrid nanosystems for efficiently treating multidrug resistant infection, skin‐tumor therapy, and enhancing wound healing. Adv Funct Mater 2019;29:1806883.

[6] Dai T , Wang C, Wang Y, Xu W, Hu J, Cheng Y. A nanocomposite hydrogel with potent and broad-spectrum antibacterial activity. ACS Appl Mater Interfaces 2018;10:15163–73. 29648438 10.1021/acsami.8b02527

[7] Zhao H , Liu M, Zhang Y, Yin J, Pei R. Nanocomposite hydrogels for tissue engineering applications. Nanoscale 2020;12:14976–95. 32644089 10.1039/d0nr03785k

[8] Lavrador Pe , Esteves MR, Gaspar VM, Mano JF. Stimuli‐responsive nanocomposite hydrogels for biomedical applications. Adv Funct Mater 2021;8:2005941.

[9] Hu J , Altun I, Zhang Z, Albadawi H, Salomao M, Mayer J, Hemachandra L, Rehman S, Oklu R. Nanocomposite hydrogels: bioactive‐tissue‐derived nanocomposite hydrogel for permanent arterial embolization and enhanced vascular healing. Adv. Mater 2020;33:202070248. 10.1002/adma.202002611 PMC7491606 32578337

[10] Mittal M , Siddiqui MR, Tran K, Reddy SP, Malik AB. Reactive oxygen species in inflammation and tissue injury. Antioxid Redox Signal 2014;20:1126–67. 23991888 10.1089/ars.2012.5149 PMC3929010

[11] Schäfer M , Werner S. Oxidative stress in normal and impaired wound repair. Pharmacol Res 2008;58:165–71. 18617006 10.1016/j.phrs.2008.06.004

[12] Koo M , Hong SH, Lee MH, Kwon B, Seon GM, Kim MS, Kim D, Nam KC, Park J. Effective stacking and transplantation of stem cell sheets using exogenous ROS-producing film for accelerated wound healing. Acta Biomater 2019;95:418–26. 30660002 10.1016/j.actbio.2019.01.019

[13] Roy S , Khanna S, Nallu K, Hunt TK, Sen CK. Dermal wound healing is subject to redox control. Mol Ther 2006;13:211–20. 16126008 10.1016/j.ymthe.2005.07.684 PMC1389791

[14] Tsang CK , Liu Y, Thomas J, Zhang Y, Zheng XFS. Superoxide dismutase 1 acts as a nuclear transcription factor to regulate oxidative stress resistance. Nat Commun 2014;5:3446. 24647101 10.1038/ncomms4446 PMC4678626

[15] Xu Z , Han S, Gu Z, Wu J. Advances and impact of antioxidant hydrogel in chronic wound healing. Adv Healthcare Mater 2020;9:1901502. 10.1002/adhm.201901502 31977162

[16] Zhang S , Ou Q, Xin P, Yuan Q, Wang Y, Wu J. Polydopamine/puerarin nanoparticle-incorporated hybrid hydrogels for enhanced wound healing. Biomater Sci 2019;7:4230–6. 31393463 10.1039/c9bm00991d

[17] Cui H , Cui L, Zhang P, Huang Y, Wei Y, Chen X. *In situ* electroactive and antioxidant supramolecular hydrogel based on cyclodextrin/copolymer inclusion for tissue engineering repair. Macromol Biosci 2014;14:440–50. 24821672 10.1002/mabi.201300366

[18] Kang B , Vales TP, Cho B, Kim J, Kim H. Development of Gallic Acid-Modified Hydrogels Using Interpenetrating Chitosan Network and Evaluation of Their Antioxidant Activity. *Molecules* 2017;22:1976. 29140278 10.3390/molecules22111976 PMC6150364

[19] Luca MD , Curcio M, Valli E, Cirillo G, Voli F, Butini ME, Farfalla A, Pantuso E, Leggio A, Nicoletta FP, Tavanti A, Iemma F, Vittorio O. Combining antioxidant hydrogels with self-assembled microparticles for multifunctional wound dressings. J Mater Chem B 2019;7:4361–70.

[20] Xue Y , Luan Q, Yang D, Yao X, Zhou K. Direct evidence for hydroxyl radical scavenging activity of cerium oxide nanoparticles. J Phys Chem C 2011;115:4433–8.

[21] Wu H , Li F, Wang S, Lu J, Li J, Du Y, Sun X, Chen X, Gao J, Ling D. Ceria nanocrystals decorated mesoporous silica nanoparticle based ROS-scavenging tissue adhesive for highly efficient regenerative wound healing. Biomaterials 2018;151:66–77. 29078200 10.1016/j.biomaterials.2017.10.018

[22] Hirst SM , Karakoti AS, Tyler RD, Sriranganathan N, Seal S, Reilly CM. Anti‐inflammatory properties of cerium oxide nanoparticles. Small 2009;5:2848–56. 19802857 10.1002/smll.200901048

[23] Chen J , Patil S, Seal S, McGinnis JF. Rare earth nanoparticles prevent retinal degeneration induced by intracellular peroxides. Nat Nanotechnol 2006;1:142–50. 18654167 10.1038/nnano.2006.91

[24] Liu T , Zhang X, Ke B, Wang Y, Wu X, Jiang G, Wu T, Nie G. F-127-PEI co-delivering docetaxel and TFPI-2 plasmid for nasopharyngeal cancer therapy. Mater Sci Eng C Mater Biol Appl 2016;61:269–77. 26838850 10.1016/j.msec.2015.12.049

[25] Akash MSH , Rehman K. Recent progress in biomedical applications of Pluronic (PF127): pharmaceutical perspectives. J Control Release 2015;209:120–38. 25921088 10.1016/j.jconrel.2015.04.032

[26] Ye F , Yaghmur A, Jensen H, Larsen SW, Larsen C, Østergaard J. Real-time UV imaging of drug diffusion and release from Pluronic F127 hydrogels. Eur J Pharm Sci 2011;43:236–43. 21550399 10.1016/j.ejps.2011.04.015

[27] Mai H , Sun L, Zhang Y, Si R, Feng W, Zhang H, Liu H, Yan C. Shape-selective synthesis and oxygen storage behavior of ceria nanopolyhedra, nanorods, and nanocubes. J Phys Chem B 2005;109:24380–5. 16375438 10.1021/jp055584b

