# Supplementary material for: Injectable self-healing ceria-based nanocomposite hydrogel with ROS-scavenging activity for skin wound repair
Source: Regen Biomater. 2021 Dec 24;9:rbab074. doi: 10.1093/rb/rbab074 (PMC9017367; doi:10.1093/rb/rbab074)
Supplement: rbab074_Supplementary_Data [file rbab074_supplementary_data.docx]

**Supporting information**

**Injectable Self-Healing Ceria-based Nanocomposite Hydrogel with ROS-scavenging Activity for Skin Wound Repair**

Xueyun Gong ^a, b^* ^#^, Meng Luo ^b^, Min Wang ^b^, Wen Niu ^b^, Yidan Wang ^b^, Bo Lei ^b^

*^a^ School of Medicine, Henan Polytechnic University, Jiaozuo 454000, China*

*^b^ Frontier Institute of Science and Technology, Xi’an Jiaotong University, Xi’an 710054, PR China*

* To whom correspondence should be directed

E-mail: [gxy@hpu.edu.cn](mailto:gxy@hpu.edu.cn)

^#^ These authors contributed equally to this work.

**Experimental section**

**1. Preparation and characterization of CeO_2_ nanorods**

The CeO_2_ nanorods was prepared similar to the previous literature.^[1]^ The details are as follows: 0.2604 g Ce(NO_3_)_3_·6H_2_O is dissolved in 1.5 ml of distilled water, and 4.32 g NaOH is dissolved in 10.5 ml of distilled water. The two solutions were mixed, stirred at room temperature for 30 min, and transferred to a 25 ml ptfe kettle. Then put it in an oven and reacted at 100 ºC for 24 h. Cooled to room temperature, centrifuged, washed with water and ethanol to obtain precipitate Ce(OH)_3_. CeO2 was obtained by drying overnight in a 60 °C oven. The morphology and structure of CeO_2_ were characterized by TEM and XRD, and the results were shown in Figure S1.

**2. Synthesis and characterization of F127-CHO**

Dibenzaldehy determinated F127 (F127-CHO) was prepared similar to the previous literature.^[2]^ Briefly, 0.5 mmol of F127 and 5 mmol of triethylamine were dissolved in 100 ml of anhydrous dichloromethane, and then 10 mmol of p-toluenesulfonyl chloride (TsCl, J&K Scientific) was added gradually. After 48 h at room temperature, the mixture was extracted by hydrochloric acid (HCl) and sodium bicarbonate (NaHCO_3_). The F127 sulfanilic acid ester (F127-TsCl) was precipitated by diethyl ether and dried under vacuum. The average yield of F127-TsCl was about 80%. The structure of F127-Tscl is determined by nuclear magnetism, as shown in Figure S2A. 2.5 g (0.2 mmol) F127-TsCl was dissolved in 50 ml DMF, 4-hydroxybenzaldehyde (0.11 g, 0.9 mmol) and K_2_CO_3_ (0.12 g, 0.9 mmol) were added followed. The mixture was stirred at 80 °C for 72 h and then cooled to room temperature. The reaction solution was extracted with CH_2_Cl_2_ after adding 50 ml H_2_O. The organic layer was dried over MgSO_4_, concentrated and precipitated in cold diethyl ether (at ten times excess). After filtration, F127 -CHO was dried at room temperature under vacuum for 24 h (Yield: 90%). The structure of F127-CHO is determined by nuclear magnetism, as shown in Figure S2B.

3.Evaluation of antioxidant performance

(1) Determination of hydroxyl radical scavenging ability of nanocomposite hydrogel by ultraviolet spectrophotometry

The hydroxyl radical scavenging ability of nanocomposite hydrogel was investigated using a similar method previously reported.^[3]^ The stock solution of nanocomposite hydrogel FVEC-1 was prepared with CeO_2_ concentration of 10 uM by dispersing in 0.1 M Tris-HCl buffer ( pH4.7). The reaction solution for photometric determination contained 1.0×10^-5^ M MV, 0.15 mM FeSO_4_, 1.0 M H_2_O_2_, 0.1 M Tris-hcl buffer (pH 4.7), and appropriate nanocomposite hydrogel. Solutions at these concentrations were used without special note. After incubation for 10 min at room temperature, the absorbance of the reaction solution was measured at 592 nm using a UV-visible spectrophotometer (Lambda 950, PerkinElmer).

(2) SOD mimetic activity assay

The superoxide anion scavenging activity of nanocomposite hydrogel was measured with SOD assay kit-WST. Briefly, 20 ml of nanocomposite hydrogel contained different Ce element concentrations (0, 0.01, 0.03, 0.06, 0.09, 0.12 mM) were incubated with assay reagent containing xanthine, xanthine oxidase, and a water-soluble tetrazolium salt, WST-1. Then the absorbance at 450 nm was recorded using a microplate reader (SpectraMax Paragigm, Molecular Devices, USA) and the superoxide anion inhibition rate was calculated according to the manufacture's formula.

4. Cytotoxicity and Hemocompatibility Evaluation

The cytotoxicity and hemocompatibility of hydrogels were investigated according to the reported method.^[4]^ Briefly, 10 ul hydrogel sterilized by ultraviolet radiation was added to L929 cells cultured in 96-well plate culture under standard conditions for co-culture. On day 1, 3 and 5, the cell culture medium was replaced with Alamar Blue solution and cultured for another 4 hours. The fluorescence intensity was measured with a microplate reader and the cell survival rate was calculated. Alamar Blue solution was used as blank control. In addition, cells were stained with the Live/Dead kit on Days 1, 3 and 5, and stained images were obtained using a laser confocal microscope. For hemocompatibility test, 1 ml fresh mouse blood was dispersed in 19 ml PBS buffer containing 2 mg heparin sodium. After the slow and uniform oscillation, the supernatant was centrifuged at 1500 rpm for 10 min. Then the supernatant was decanted, the remaining blood cells were washed with PBS buffer and centrifuged three times. Then, the remaining blood cells were dispersed in 20 ml PBS buffer. After that, 20 ul hydrogel was added to 200 ul blood cell dispersion and placed at 37 ℃ for 60 min. PBS and Triton X-100 were used as negative and positive controls, respectively. The absorbance at 540 nm was measured with a microplate reader, and the hemolysis rate was calculated according to the following formula:

hemolysis rate = (A_n_-A_P_)/(A_T_-A_P_)× 100%

A_n_, A_P_, and A_T_ represent the absorbance of hydrogel, negative control PBS, and positive control Triton X-100, respectively.

References

1. Mai H X, Sun L D, Zhang Y W, et al. Shape-selective synthesis and oxygen storage behavior of ceria nanopolyhedra, nanorods, and nanocubes[J]. The Journal of Physical Chemistry B, 2005, 109(51): 24380-24385.

2. Zhou L, Xi Y, Xue Y, et al. Injectable Self‐Healing Antibacterial Bioactive Polypeptide‐Based Hybrid Nanosystems for Efficiently Treating Multidrug Resistant Infection, Skin‐Tumor Therapy, and Enhancing Wound Healing[J]. Advanced Functional Materials, 2019, 29(22): 1806883.

3. Xue Y, Luan Q, Yang D, et al. Direct Evidence for Hydroxyl Radical Scavenging Activity of Cerium Oxide Nanoparticles[J]. Journal of Physical Chemistry C, 2011, 115(11): 4433-4438.

4. Xi Y, Ge J, Guo Y, et al. Biomimetic elastomeric polypeptide-based nanofibrous matrix for overcoming multidrug-resistant bacteria and enhancing full-thickness wound healing/skin regeneration[J]. ACS nano, 2018, 12(11): 10772-10784.


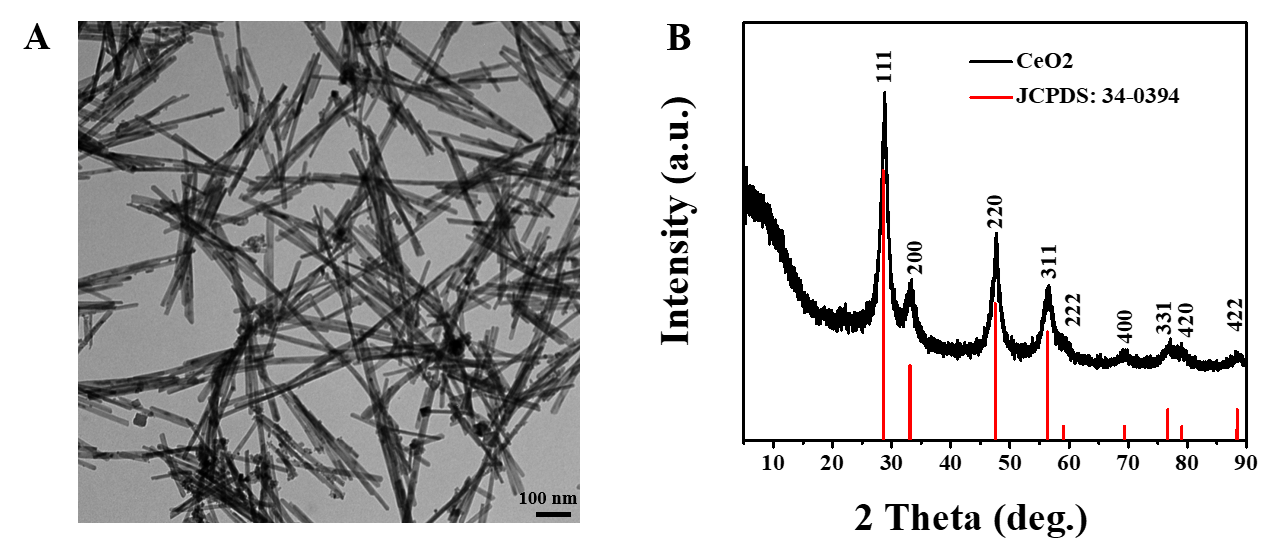


Figure S1. (A) TEM images of CeO_2_; (B) XRD analysis of CeO_2_.


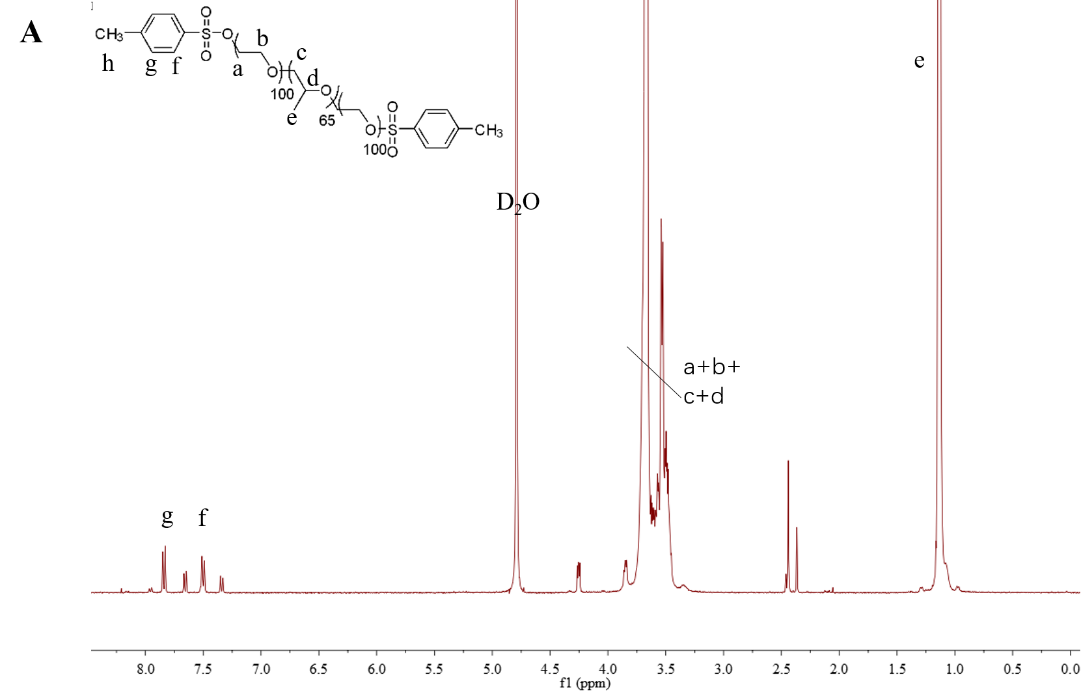


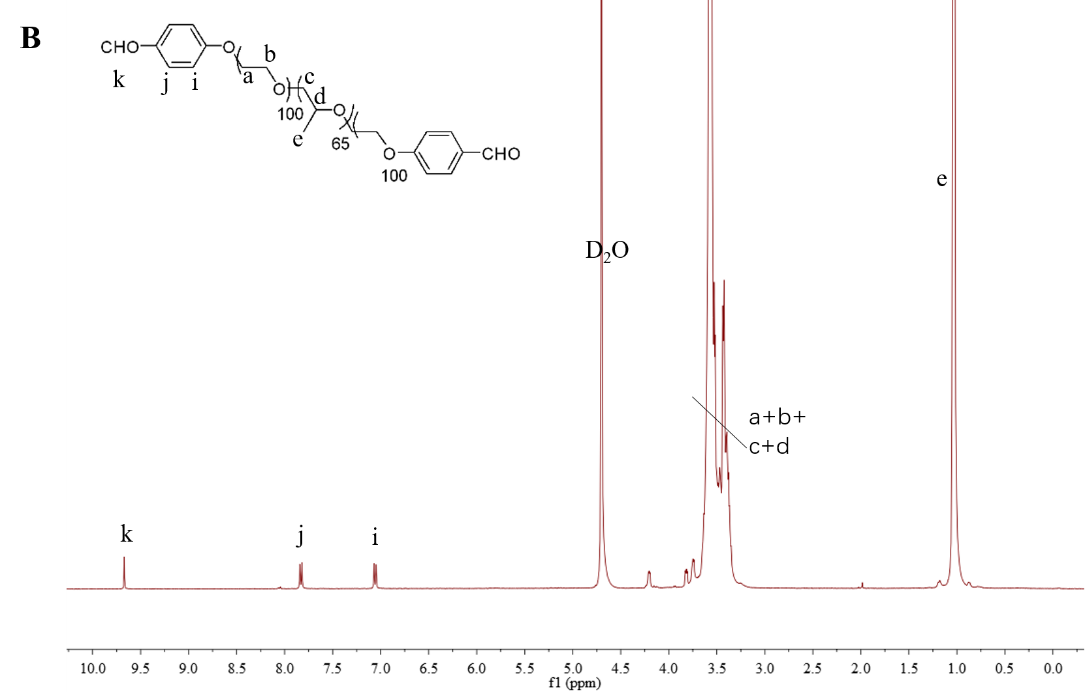


**Figure S2. ^1^ H NMR spectra of F127-TsCl (A) and F127- CHO (B) in D_2_O.**





**Figure S3A**. UV-vis absorption spectrums of 1.0×10^-5^ M MV, 0.15 mM FeSO_4_, nanocomposite hydrogel contained 10 nM CeO_2_, 1.0 mM H_2_O_2_, and the Fenton reagent including 1.0 mM H_2_O_2_ and 0.15 mM FeSO_4_ in Tris-HCl solution (pH 4.7).





**Figure S3B**. UV-vis absorption spectra of MV, MV/H_2_O_2_/nanocomposite hydrogel contained 10 nM CeO_2_, MV/FeSO_4_/H_2_O_2_, and MV/FeSO_4_/H_2_O_2_/ nanocomposite hydrogel contained 10 nM CeO_2_ solutions at an incubation time of 10 min.


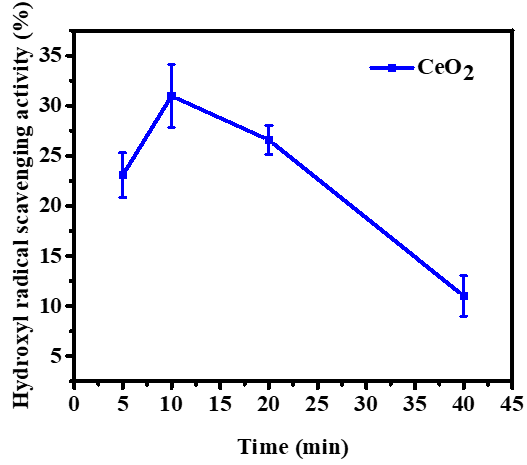


**Figure S4. The ROS scavenging ability evaluation of hydrogel time-dependent.**


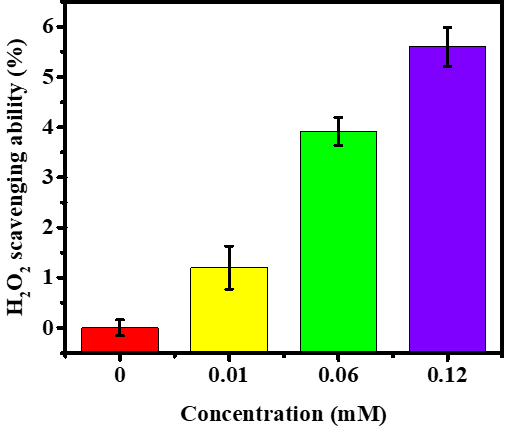


**Figure S5**. The H_2_O_2_ scavenging ability of hydrogel with different concentrations.


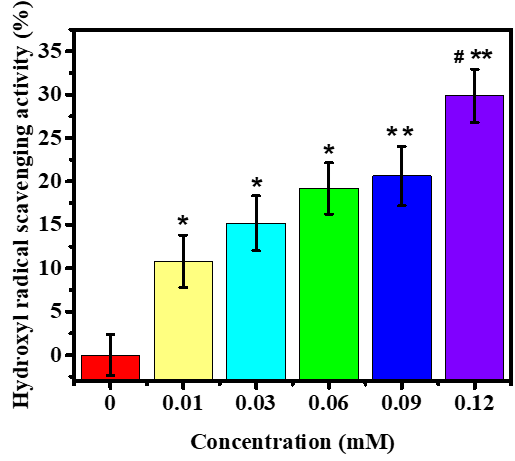


**Figure S6**. The ROS scavenging ability of CeO_2_ with different concentrations.


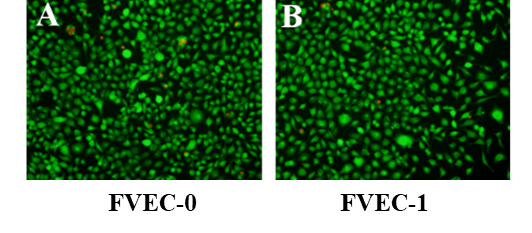


**Figure S7. Live-Dead L929 cell staining images after incubated with FVEC hydrogel for 5 days.**
